# Versatile sample environments and automation for biological solution X-ray scattering experiments at the P12 beamline (PETRA III, DESY)

**DOI:** 10.1107/S160057671500254X

**Published:** 2015-03-12

**Authors:** Clement E. Blanchet, Alessandro Spilotros, Frank Schwemmer, Melissa A. Graewert, Alexey Kikhney, Cy M. Jeffries, Daniel Franke, Daniel Mark, Roland Zengerle, Florent Cipriani, Stefan Fiedler, Manfred Roessle, Dmitri I. Svergun

**Affiliations:** a European Molecular Biology Laboratory, Hamburg Outstation, Notkestrasse 85, Hamburg, 22603, Germany; b Laboratory for MEMS Applications, IMTEK – Department of Microsystems Engineering, University of Freiburg, Georges-Koegler-Allee 103, Freiburg, 79110, Germany; c European Molecular Biology Laboratory, Grenoble Outstation, 6 rue Jules Horowitz, BP 181, Grenoble, 38042, France

**Keywords:** biological small-angle X-ray scattering, high-brilliance P12 synchrotron beamline, automated hardware and software systems

## Abstract

A high-brilliance synchrotron P12 beamline of the EMBL located at the PETRA III storage ring (DESY, Hamburg) is dedicated to biological small-angle X-ray scattering (SAXS) and has been designed and optimized for scattering experiments on macromolecular solutions. Scatterless slits reduce the parasitic scattering, a custom-designed miniature active beamstop ensures accurate data normalization and the photon-counting PILATUS 2M detector enables the background-free detection of weak scattering signals. The high flux and small beam size allow for rapid experiments with exposure time down to 30–50 ms covering the resolution range from about 300 to 0.5 nm. P12 possesses a versatile and flexible sample environment system that caters for the diverse experimental needs required to study macromolecular solutions. These include an in-vacuum capillary mode for standard batch sample analyses with robotic sample delivery and for continuous-flow in-line sample purification and characterization, as well as an in-air capillary time-resolved stopped-flow setup. A novel microfluidic centrifugal mixing device (SAXS disc) is developed for a high-throughput screening mode using sub-microlitre sample volumes. Automation is a key feature of P12; it is controlled by a beamline meta server, which coordinates and schedules experiments from either standard or nonstandard operational setups. The integrated SASFLOW pipeline automatically checks for consistency, and processes and analyses the data, providing near real-time assessments of overall parameters and the generation of low-resolution models within minutes of data collection. These advances, combined with a remote access option, allow for rapid high-throughput analysis, as well as time-resolved and screening experiments for novice and expert biological SAXS users.

## Abstract 2

An integrated environment for biological small-angle X-ray scattering (BioSAXS) at the high-brilliance P12 synchrotron beamline of the EMBL (DESY, Hamburg) allows for a broad range of solution scattering experiments. Automated hardware and software systems have been designed to ensure that data collection and processing are efficient, streamlined and user friendly.

## 1. Introduction   

Small-angle X-ray scattering (SAXS) is an experimental technique that is used to extract global structural parameters and shape information in the scale ranging from nanometres to a micrometre. Every material with inhomogeneous electron density can be probed using SAXS, which is routinely employed to probe the structures of polymers (Chu & Hsiao, 2001 ▶), tissues (Paris *et al.*, 2000 ▶; Fernández *et al.*, 2002 ▶), alloys and ceramics (Fratzl, 2003 ▶) as well as biological macromolecules and nanocomposites in solution (Blanchet & Svergun, 2013 ▶; Spilotros & Svergun, 2014 ▶). The scattering from solutions is usually isotropic owing to the random position and orientation of particles, and the scattering intensity *I*(*s*) is presented as a function of momentum transfer *s* = 4πsinθ/λ, where 2θ is the scattering angle and λ is the wavelength of the X-rays. Biomacromolecular solution SAXS presents unique experimental challenges. Proteins, nucleic acids, lipids and carbohydrates have low contrasts relative to their typically aqueous solvents (*i.e.* the difference in electron density between the dissolved particles and the solvent is small). Consequently, the solute scattering intensities obtained after subtraction of the solvent scattering are generally very weak. Moreover, pure and stable biological samples can be difficult to produce in quantity, and these often precious samples can be potentially affected by X-ray radiation damage, resulting in the production of aggregates that spoil measurements. Dedicated synchrotron BioSAXS beamlines must take these complications into account and balance the advantages afforded by high-brilliance sources that open new avenues of structural investigation – such as high-throughput screening operations, in-line component separation, real-time kinetics and microfluidics – with the constraints imposed by the fragility and availability of biological samples.

Recently developed approaches to extract rich structural information from SAXS data on a broad range of molecule sizes and experimental conditions have led to a dramatically increased popularity of solution SAXS as both a standalone and complementary tool for structural biologists (Lipfert & Doniach, 2007 ▶; Graewert & Svergun, 2013 ▶). Several beamlines partly or entirely dedicated to BioSAXS have been built over the past few years (David & Pérez, 2009 ▶; Classen *et al.*, 2010 ▶; Blanchet *et al.*, 2012 ▶; Nielsen *et al.*, 2012 ▶; Kirby *et al.*, 2013 ▶; Labrador *et al.*, 2013 ▶; Pernot *et al.*, 2013 ▶). Many of these instruments have been engineered to optimize the collection of weak scattering signals in combination with new sample handling environments and devices for specialized experiments. The EMBL X33 beamline located on the DORIS III storage ring (DESY, Hamburg) was one of the first SAXS beamlines entirely dedicated to the analysis of biological macromolecules. The significant advances in hardware and in software made at X33 (Roessle *et al.*, 2007 ▶; Blanchet *et al.*, 2012 ▶) have been transferred to the new P12 BioSAXS beamline at the storage ring PETRA III (synchrotron DESY, Hamburg). The beamline constructed and run by EMBL in Hamburg with a Collaborative Access Group contribution from the Helmholtz-Zentrum Geesthacht (HZG) has now been in full operation since 2013 with an extensive user program. P12 utilizes a focused monochromatic X-ray beam from an undulator source and offers high brilliance and reduced background coupled with automated sample handling, data collection and processing protocols for a variety of solution scattering experiments. This manuscript is devoted to describing the automation and possibilities available at P12 for performing various types of biological solution scattering experiments. In addition to a fully automated and remote high-throughput operation with a robotic sample changer, sample environments and control systems have been developed for in-line size-exclusion chromatography component separation with additional detectors, as well as a stop-and-flow device for fast-time kinetics. A novel microfluidic system for ultra-low sample volume handling and mixing, the SAXS disc, has been developed for concentration, additive or stability screening.

## 2. P12 beamline overview   

In this section, we shall give a brief summary of the design and of the relevant characteristics of P12, as well as presenting more specific developments making the beamline especially suitable for the solution scattering experiments. Monochromatic X-rays of the P12 BioSAXS beamline are sourced from the controllable-gap low-divergence U29 undulator of PETRA III. A double-crystal Si(111) monochomator allows tuneable energy selections between 4 and 20 keV (λ = 0.06–0.3 nm), with a typical default energy of 10 keV (λ = 0.124 nm) used for most operations. Adaptive bimorph mirrors in a Kirkpatrick–Baez geometry combined with a three-pair slit collimation produce a close to one-to-one optics that conserves the low divergence of the source, delivering an intense (10^13^ photons s^−1^) highly focused small beam to the sample position (120 × 200 µm full width at half-maximum). The entire beamline (source-to-detector length of 83 m), including an adjustable detector tube (sample–detector distance of 1.6, 2.1, 3.1, 4.1 or 6.1 m; usually 3.1 m), remains under vacuum for standard quartz capillary sample cell measurements. Any nonstandard sample environment can be positioned into the beam using an in-air configuration, with Kapton sealing windows across an air gap of a few centimetres, aided by motorized *x*–*y* sample stages and an on-axis camera.

For biological solution scattering experiments the fraction of photons scattered by the solutes is exceptionally low, of the order of 10^−6^ of the incident photons (Stuhrmann, 1980 ▶). In order to properly extract the weak scattering signal from the target of interest it is necessary that (*a*) the scattered photons are collected with a low-noise detector, (*b*) parasitic scattering from the optical components of the beamline is minimized, (*c*) the data are accurately normalized to the beam intensity and transmittance of the sample, and (*d*) means are available to reduce the effects of radiation damage. Below, solutions implemented at P12 to meet these requirements are briefly described.

### 2.1. Detector   

The P12 beamline is equipped with a hybrid photon-counting pixel detector (Henrich *et al.*, 2009 ▶) having practically no detector readout noise, a high (20 bits) dynamic range and a short (few ms) readout time. The Pilatus 2M detector (Dectris), consisting of 1475 × 1679 pixel (172 × 172 µm) modular arrays with a total active area of 253.7 × 288.8 mm, is mounted on railings for motorized translation between the five positions spanning 1.6–6.1 m. The detector can be offset such that at the default position of 3.1 m, at an energy of 10 keV (λ = 0.124 nm), the SAXS data are recorded in the range *s* = 0.02–4.5 nm^−1^, corresponding to a real-space resolution of 300–1.5 nm. By varying the sample–detector distance and, if necessary, the beam energy momentum transfers down to *s* = 0.006 nm^−1^ and up to 16 nm^−1^ are reached (resolution from 1000 to 0.4 nm).

### 2.2. Reducing instrument background: scatterless slits   

**Figure 1:**
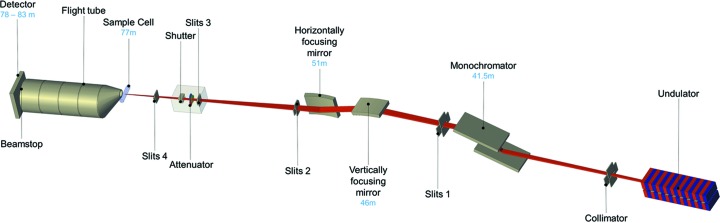
Schematic representation of the major elements of the P12 beamline. The distances of the main components from the undulator source are indicated in blue.

**Figure 2:**
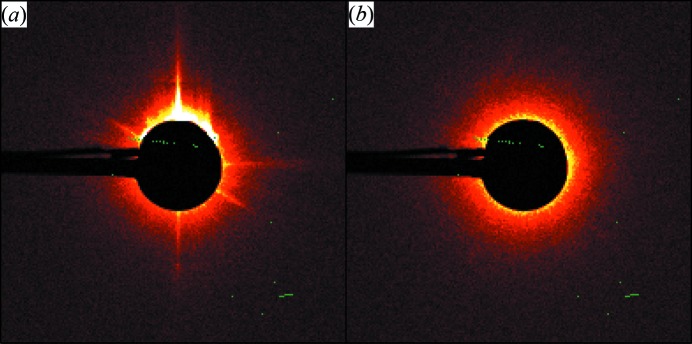
Detector images showing the parasitic scattering around the beamstop with (*a*) conventional and (*b*) scatterless slits.

Every component in contact with the beam (the sample cell, windows, optical elements, slits and air) may scatter X-rays that will contribute to the instrument background. As biological macromolecular SAXS is contingent on the accurate subtraction of all background scattering contributions, reducing the effects of parasitic scattering is vital not only to improve signal-to-noise ratios in the data but also to remove sources of scattering that may perturb the extraction of structural parameters. The instrument background at P12 was originally reduced by a standard three-paired-slits collimator system (slits 2, 3 and 4 in Fig. 1 ▶), where (*a*) slits 2 define the beam and adjust the flux, (*b*) slits 3 cut out the parasitic scattering fan coming from the optics and passing through the first pair of slits, and (*c*) the last pair of slits (slits 4) remove the weaker secondary scattering produced by the second pair of slits. However, the last pair of guard slits can still produce strong parasitic scattering, especially at low angles (Fig. 2 ▶). To further reduce this background the last pair of guard slits has been replaced by hybrid scatterless slits. These are composed of heavy metal blades with the monocrystal lateral edges oriented far from any Bragg peak position with respect to the incident beam (Li *et al.*, 2008 ▶). The scatterless slits installed at P12 (from Xenocs, Sassenage) can sustain a higher radiation load, compared with regular guard slits, without producing significant parasitic scattering and they significantly reduce the overall instrument background (Fig. 2 ▶).

### 2.3. Accurate data normalization: a miniature active beamstop   

**Figure 3:**
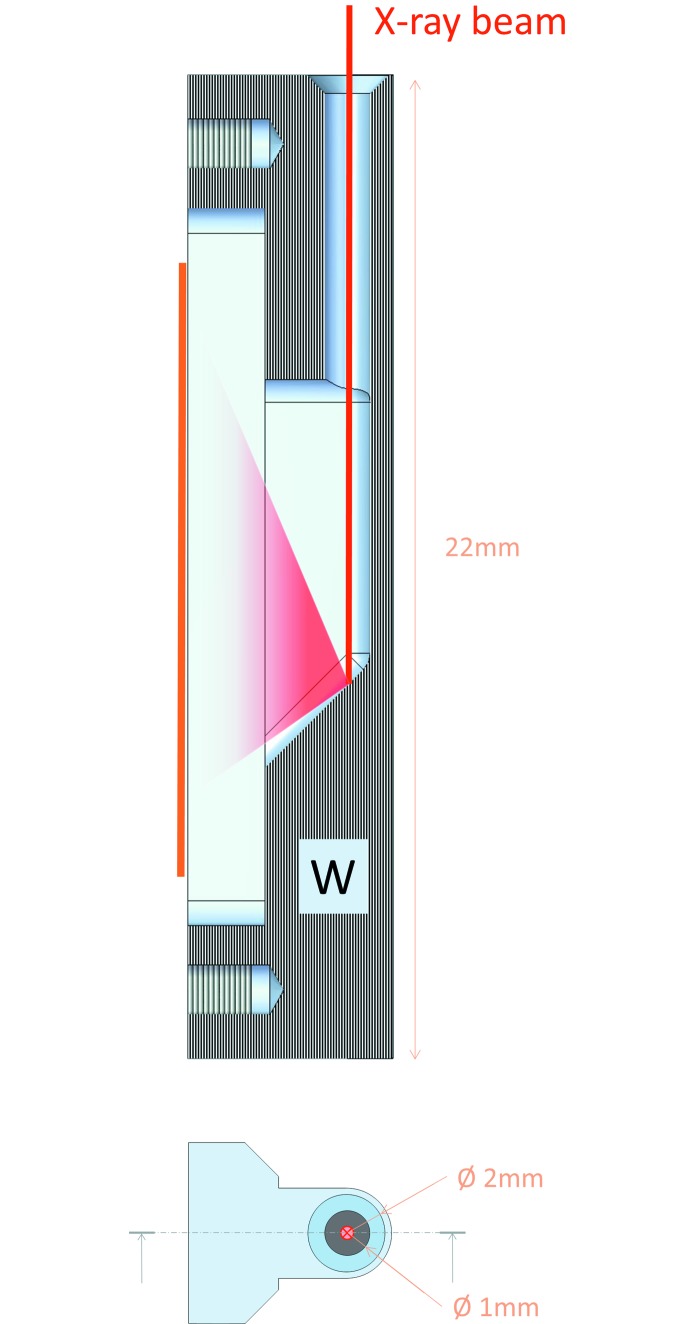
Sectional view (top) of the tungsten chamber, illustrating the principle of indirect flux monitoring. The primary beam (red) hits the back wall of the chamber where most of the photons are absorbed, but part of them are scattered towards the photosensitive area of the diode (not represented here). Front view (bottom) of the beamstop with its physical diameter and the diameter of the active area indicated.

A crucial step in solution scattering data processing is the subtraction of the solvent scattering from that of the sample, and these two independent patterns must be appropriately normalized to take into account possible variation in the beam flux and differences in transmission. This operation can be conveniently accomplished by using an active beamstop. The principal function of a beamstop is to prevent the primary beam from hitting the detector. Moreover, the beamstop position in front of the detector also provides an optimal place to measure the transmitted beam flux during the course of exposure. Division of the measured intensities by the monitor of the transmitted beam allows one to account for both the incoming flux and transmission differences. A new miniature 2 mm-diameter active beamstop has been developed and installed on P12. The accurate estimation of the beam flux is based on an indirect detection of X-rays using a pin diode (Fig. 3 ▶) as opposed to direct pin diode X-ray measurements (Ellis *et al.*, 2003 ▶). Taking the indirect approach significantly extends the diode lifetime and overcomes the size limitations of classical pin diode beamstops, thus allowing one to register smaller angles at the given detector position. Specific details of the new miniature beamstop technology are presented by Blanchet *et al.* (2015 ▶).

### 2.4. Reducing radiation damage: beam attenuators   

Radiation damage to biological samples resulting in the formation of aggregates can cause significant problems at high-brilliance synchrotron X-ray sources. The P12 beamline is equipped with a beam attenuator composed of three filter wheels, each wheel containing ten holders for attenuating foil. Nine of these holders house attenuation foils of different thicknesses (aluminium foil of 6–54 µm for the first wheel, and of 60–540 µm for the second wheel, and titanium foil of 75–675 µm for the last wheel) and one holder is kept free. Different combinations of the wheel rotations allow a total of 1000 options of the degree of attenuation (*e.g.* at 10 keV, from a factor of 0.96 to 2.10^−15^) to be selected. Beam attenuation can thus be finely tuned and tailored to the properties of specific samples to achieve optimal balance between lowering the useful signal and reducing the effects of radiation damage (Jeffries *et al.*, 2015 ▶).

## 3. Dedicated sample environments for in-vacuum measurements   

As virtually any element in the path of the beam is a source of parasitic scattering the ideal sample holder should be placed in vacuum. In-vacuum sample holders avoid the scattering from the gas molecules in the air gap and eliminate the X-ray absorption and scattering by the extra windows, thus reducing the background scattering (Dubuisson *et al.*, 1997 ▶). In this section, the sample environments allowing for in-vacuum measurements are described.

### 3.1. Measurement cell   

The standard SAXS sample cell configuration at P12 consists of a horizontal thermostated (278–323 K) quartz capillary with 50 µm-thick walls and a 1.7 mm path length housed in a specialized stainless steel pod for in-vacuum measurements. Quartz exhibits good mechanical stability in vacuum with inherently low X-ray absorption and scattering properties that in combination with the in-vacuum setup improve significantly the signal-to-noise ratio in the data. Quartz is chemically stable and bio-inert towards most biological macromolecules and their solution buffers and is thus an ideal material for housing a diverse range of samples. The optimal sample thickness varies depending on the energy [*e.g.* at 8 keV, the optimal path length is 1 mm (Glatter & Kratky, 1982 ▶)]; however, for the standard BioSAXS experiments performed at P12 at high flux, the 1.7 mm standard cell (optimal for 9.5 keV photons) suffices for most data collection requirements between 6 and 20 keV.

The stainless steel pod that houses the capillary has been designed to be easily changeable in case of a leak or irreversible capillary fouling. The universal adaptors of the pod allow for the connection of multiple sample delivery devices, enabling both manual and automatic sample loading. Typically, samples (with a volume between 20 and 35 µl) are delivered to the capillary for continuous-flow SAXS measurements using a third-generation EMBL/ESRF sample changer (see below). Continuous flow significantly reduces the effects of X-ray radiation damage (Lipfert *et al.*, 2006 ▶). Automated loading of very low sample volumes (5 µl) for static sample data collection is also possible as well as manual sample loading *via* syringe or using alternative sample delivery systems, for example, the eluent from size-exclusion columns used for component separation experiments (see below).

### 3.2. Batch-mode analysis: the EMBL/ESRF automated sample changer   

The first use of sample-changer robots on a SAXS beamline was less than a decade ago, and many modern SAXS facilities are now equipped either with adapted commercial products (David & Pérez, 2009 ▶; Classen *et al.*, 2010 ▶; Kirby *et al.*, 2013 ▶) or with devices specifically developed for solution SAXS (Round *et al.*, 2008 ▶, 2015 ▶; Blanchet *et al.*, 2012 ▶; Nielsen *et al.*, 2012 ▶). The P12 automated sample-changer robot was built as a collaborative effort between EMBL-Hamburg, EMBL-Grenoble and ESRF. This machine has been designed for BioSAXS experiments, specifically tailored for high-throughput handling and for a controlled delivery of samples into the exposure cell. Here, we give an overview of the robot and its integration in the beamline; a detailed description is presented elsewhere (Round *et al.*, 2015 ▶).

Samples are measured at different concentrations, typically between 0.5 and 10 mg ml^−1^, to detect interaction between the solutes. Between 10 and 40 µl of solution is required for each measurement and can be stored along with their corresponding buffer in the sample changer. The sample storage plate accommodates PCR-tube strips (4 × 8 × 200 µl tubes), 1.5 ml Eppendorf tubes, 96 well plates and 96 sample deep-well blocks. In total, more than 200 samples can be stored in the plate. The block is temperature controlled and is covered with a metal capping plate to limit water condensation and sample evaporation during and between SAXS measurements. Sample volumes (5–35 µl) are transferred to the SAXS capillary using a polytetrafluoroethylene-lined needle driven by a precision syringe pump. Samples are loaded using built-in protocols that drive the sample storage plate to the fixed-position needle as opposed to moving the needle to the sample plate. This design ensures that the length of the tubing between the needle entry point and the measurement cell can be kept constant and as short as possible. We have found these conditions to be critical for consistent and reproducible sample loading into the SAXS capillary. The dead volume of the system is of the order of 1–2 µl, depending on sample adsorption. The precision pump can be programmed to perform continuous flow or static sample SAXS measurements and, if necessary, can be driven in reverse to recover samples after data acquisition.

The accurate positioning of the sample in the capillary is monitored by a camera and automated through image processing. Data acquisition is triggered by the beamline meta server software (BMS) and, for most standard operations, the sample undergoes continuous flow during the measurement. The sample flow rate is determined automatically by the amount of sample and by the total exposure time, typically divided into 20 × 50 ms exposures for a total of 1 s. After the SAXS data have been collected, the sample is either flushed to waste, or recovered, and a subsequent automated washing cycle cleans the sample capillary. The wash cycle consists of successive rapid-flow detergent–ethanol–water and MilliQ-water flushes, followed by high-pressure drying with filtered air. Capillary drying is monitored by a camera using image processing techniques to prevent residual water from the wash cycle contaminating the subsequent samples. The full cycle consisting of the sample loading, data acquisition, capillary washing and drying takes approximately 50 s. The operation of the robotic sample changer is highly reliable; during the beam year in 2013, over 85000 samples were measured and only five malfunctions were detected.

### 3.3. Online sample purification and characterization   

A batch mode using a robotic sample changer is generally applicable for most BioSAXS solution scattering experiments. However, some samples may have an inherent tendency to aggregate or oligomerize over time and consequently exist as mixtures of components in solution. As the X-ray scattering by a particle scales with the square of its volume, the presence of even small quantities of aggregates or oligomers can significantly complicate data analysis and interpretation. The separation of aggregates from a target of interest, or the separation of oligomeric mixtures into their respective individual components, is possible at P12 using size-exclusion chromatography (SEC). [For examples of other SEC–SAXS setups, see Mathew *et al.* (2004 ▶), David & Pérez (2009 ▶), Kirby *et al.* (2013 ▶) and Round *et al.* (2013 ▶).]

Between 50 and 100 µl of sample at 5–10 mg ml^−1^ are manually injected onto a selected analytical SEC column (1–24 ml, *e.g.* a Superdex 200 Increase 10/300 GL) and pumped through the column under continuous flow (0.4 ml min^−1^) using an isocratic pump (Viscotek VE-1122). Although the sample is diluted in the chromatography column, meaningful SAXS data can be acquired from the sample components as they elute from the column matrix by connecting the output to the sample capillary. The SAXS data collected before the solutes flow out of the column can be used for matched buffer subtraction. At P12, the SEC–SAXS experiments can additionally be combined with a triple detector array (TDA 305, Malvern Instruments) consisting of right-angle laser light scattering (RALLS), refractive index (RI) and ultraviolet (UV) detectors. This output can then be correlated with the SAXS forward scattering intensities [*I*(0)] to cross-validate the molecular weights (MW) of the eluting species.

#### 3.3.1. SEC–SAXS example: ovalbumin   

**Figure 4:**
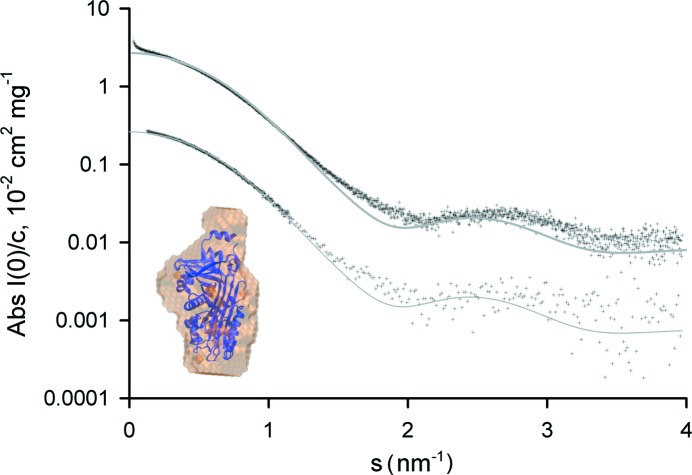
Experimental SAXS curves of ovalbumin collected with the sample changer (top) and with online SEC (bottom; offset by one order of magnitude). The online SEC curve is noisier than the sample changer curve because of the much lower solute concentration, and the higher angle data (for *s* > 1.5 nm^−1^) have been re-binned with a binning factor of five for better visualization. The grey lines represent in both cases the fits by the curve computed from the atomic structure. Inset: *ab initio* model of the ovalbumin monomer (orange) built from the SEC–SAXS experimental curve; the aligned atomic structure is represented in blue for comparison.

The advantages offered by SEC–SAXS are illustrated in Fig. 4 ▶, where ovalbumin data obtained from batch-mode SAXS and SEC–SAXS are compared. Chicken ovalbumin was acquired from GE Healthcare. For SEC–SAXS/TDA analysis the samples were dissolved in 100 m*M* Tris pH 7.5, 150 m*M* NaCl, 5% glycerol at a concentration of 15 mg ml^−1^. The sample was filtered through a 0.2 µm centrifugal filter unit (Millipore) prior to loading on to the Superdex 200 10/300 column. The final solute concentrations were determined by measuring the absorbance at 280 nm (Thermo Scientific NanoDrop ND-1000) and using calculated extinction coefficients expressed as ∊_1%_ (10 mg ml^−1^) from ProtParam: ∊_ovalbumin_ = 7.32. A clear misfit is observed between the experimental scattering obtained in batch mode and the scattering calculated using *CRYSOL* (Svergun *et al.*, 1995 ▶) from the crystallographic monomer of the protein [Protein Data Bank (PDB) code 1ova (Stein *et al.*, 1991 ▶)], yielding a discrepancy χ^2^ = 5.6. Notably, at low angles the experimental scattering intensity is systematically higher than that of the model, and the experimental radius of gyration *R*_g_ significantly exceeds the model one (3 nm *versus* 2.5 nm, respectively) suggesting that larger species exist in the sample, presumably consisting of ovalbumin oligomers.

**Figure 5:**
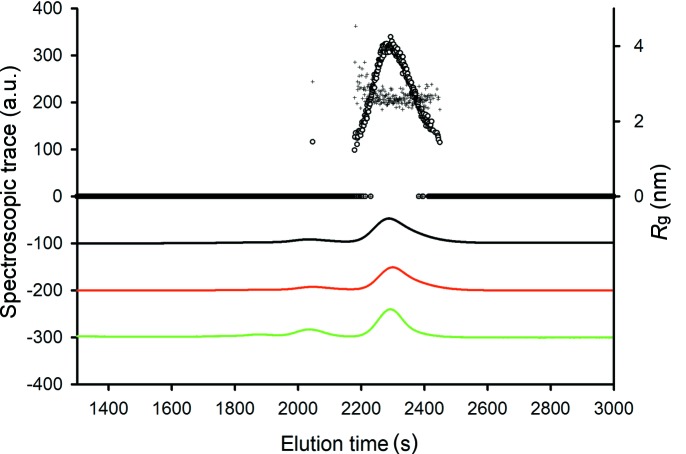
Spectroscopic signal and SAXS-derived parameters collected after the size-exclusion column. The black line corresponds to the UV signal, the red line corresponds to the refractive index and the green line corresponds to the right-angle light scattering. The signals are in arbitrary units and have been offset for clarity. Forward scattering (○) and radius of gyration (+) were computed from the buffer subtracted curves.

In addition to the SEC–SAXS data, the TDA setup was used for a further characterization of the sample (Fig. 5 ▶). The RI/RALLS/UV elution trace displays the main peak after 2300 s, which correlates well with the expected MW of ovalbumin monomers (MW_RALLS_ = 47.8 kDa; MW_expected_ = 43 kDa) and a weaker peak corresponding to an ovalbumin dimer. Each SAXS frame collected is processed as described in §5.3 and the radius of gyration and forward scattering computed from the different frames are also plotted in Fig. 5 ▶ against the elution time. The traces of *I*(0) and *R*_g_ are consistent with the other spectroscopic traces, although the multimer is hardly detected by SAXS because of its low concentration. The automated averaging of the individual SAXS data frames spanning the peak produces a final SAXS profile yielding an *R*_g_ value of 2.5 nm, in agreement with the *R*_g_ value computed from the monomer atomic structure. Furthermore, the calculated model intensity from the crystal structure (PDB ID: 1ova) shows a significant improvement in the fit against the experimental data (χ^2^ = 2.02). A low-resolution *ab initio* bead model automatically generated by the pipeline using *DAMMIF* models for initial search and *DAMMIN* for refinement (Svergun, 1999 ▶; Kozin & Svergun, 2001 ▶; Volkov & Svergun, 2003 ▶; Franke & Svergun, 2009 ▶) aligns well with the previously solved crystal structure (Fig. 4 ▶). This example illustrates the utility of SEC–SAXS with TDA for extracting individual component scattering data from polydisperse samples. Although a SEC–SAXS/TDA run takes significantly more time than a batch-mode sample changer measurement (about 0.5–1 h *versus* 3 min) and requires higher sample concentrations and volumes, the technique is extremely valuable for the analysis of complex systems.

## 4. Experimental setups for nonstandard SAXS experiments   

The in-vacuum capillary is an optimal setup for automated, high-throughput and on-line purification studies of solutions, but for certain types of experiment this setup is not feasible. It may be necessary, for example, to perform experiments in solution with aggressive chemicals or at extreme temperatures; gels and tissues may require specialized sample holders, *etc*. This section presents some of the nonstandard experiments performed at P12 using specifically tailored in-air sample environments. These include, but are not limited to, high-temperature investigations, stopped-flow time-resolved kinetic experiments, and a microfluidic sample screening platform, the SAXS disc, for the rapid mixing and analysis of sub-microlitre sample volumes.

### 4.1. The in-air setup   

The sample-changer robot and the SAXS capillary cell can be removed as a single module from the P12 beamline and replaced with virtually any in-air sample environment. Extendable vacuum pipes sealed with Kapton windows are attached to the beamline to minimize the air gap around the sample position and to keep the background scattering from air to a minimum. The experimental table on which these sample environments are placed can support devices of up to 500 kg; for example, a high-temperature-sample holder can be mounted and aligned using an *x*–*z* linear translation stage with micrometre precision. A customized in-house on-axis microscope provides a magnified image of the sample position in the direction of the incident beam without incurring parallax error, enabling precise sample holder alignment. Users may also bring their own customized sample environments; recently, devices such as a hexapod for scanning SAXS applications and a heating stage (Linkham, Tadworth, UK) to reach extreme temperatures were successfully utilized at P12 by users from HZG.

#### 4.1.1. Time-resolved SAXS environments   

In a time-resolved experiment, SAXS data are collected from the intermediate states of a system as they shift toward a new equilibrium position. Controlled triggering of a reaction can be realized in several ways, for example by mixing two solutions, by changing the temperature or the pressure, or by directly activating a system using a laser-controlled photo reaction (Nölting, 2005 ▶; Callender & Dyer, 2006 ▶). The timescales for the experiments are broad, spanning from the sub-millisecond range for rapid protein folding events (Kathuria *et al.*, 2011 ▶; Lindorff-Larsen *et al.*, 2011 ▶) to hours for amyloid fibril and assembly formation (Vestergaard *et al.*, 2007 ▶). Performing time-resolved experiments requires appropriate planning and control of the triggering mechanism based on the *a priori* information on the timescale of the reaction.

**Figure 6:**
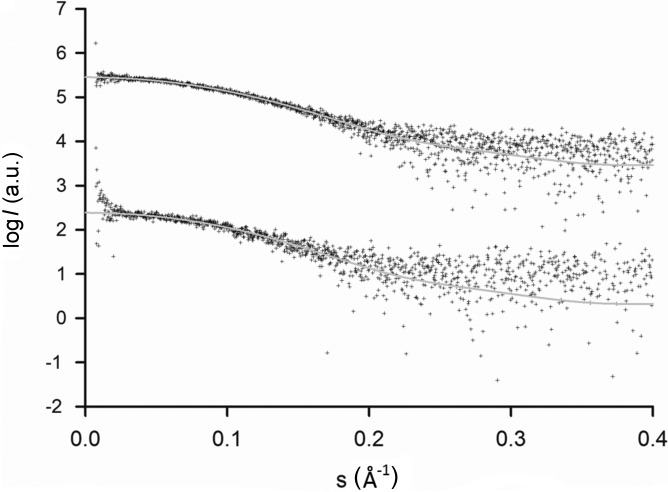
SAXS data from ribonuclease A measured in the sample changer (top) and on the disc (bottom), both with an exposure time of 50 ms, compared against the theoretical scattering computed from the atomic structure (PDB code: 1fs3). The curves are offset for clarity.

Kinetic investigations at P12 take advantage of the highly brilliant beam source and a rapid detector, both allowing for measurement times in the millisecond range. To date, most of the kinetic reactions studied on P12 have been triggered by solution mixing. The reactions on the scale of seconds can be measured in the sample changer using the standard in-vacuum configuration and do not require a dedicated experimental setup. For faster kinetics, an SFM300 Biologic stopped-flow device with a dead time of about 3 ms is mounted on the experimental table and aligned with the beam. Owing to the high flux and the low instrument background of P12, sufficient scattered photons can be collected in 30–50 ms to obtain an interpretable SAXS profile. Although the data are noisy, these measurements not only allow the radius of gyration *R*_g_ to be monitored but also permit more sophisticated analyses (an example of a typical 50 ms frame is displayed in Fig. 6 ▶). The timescale of the stopped-flow experiments fits with the repetition rate (30 Hz) of the Pilatus 2M detector, and kinetic reactions can be followed with a time resolution of 33 ms. Several P12 user groups have employed the stopped-flow system for the analysis of their samples, including the analysis of the tRNA-modifying enzyme complex MnmE/MnmG (Fislage *et al.*, 2014 ▶).

A disadvantage of the stopped-flow apparatus is in its significant sample consumption. The device alone has a dead volume of over 100 µl, and repeated measurements are often needed to improve the signal-to-noise ratio in the data or to overcome radiation damage (if detected). Consequently, up to several millilitres of sample may be required to complete an experiment, which may be a prohibitively large quantity for many biological samples.

### 4.2. Microfluidic applications: the SAXS disc   

Microfluidic devices allow for a rapid analysis of samples using a wide range of analytical techniques combined with limited sample consumption. Fabricated microfluidic chips have been used in combination with SAXS for a number of applications. These include time-resolved experiments to study kinetics in a continuous-flow chip (Graceffa *et al.*, 2013 ▶), exploration of macromolecular conformational states in a shear flow environment (Martin *et al.*, 2010 ▶; Trebbin *et al.*, 2013 ▶), rapid mixing and sample dilution to measure the effects of solute concentration (Lafleur *et al.*, 2011 ▶), and online complex sample preparation (Martel *et al.*, 2008 ▶). Although microfluidic systems are effectively miniaturized ‘labs-on-a-chip’, the accessory components often required to control and operate the devices (pumps, valves, electronics, pneumatics *etc.*) can be very bulky; thus converting the lab-on-a-chip concept into what could be described as a ‘chip-in-a-lab’. Here, we present a novel device, a centrifugal-force-driven microfluidic screening disc for solution SAXS measurements. The SAXS disc forgoes the need for any external pump or valve attachments during SAXS data collection (Ducrée *et al.*, 2007 ▶) and offers a wide range of experimental possibilities for the structural biology user community.

#### 4.2.1. SAXS disc operation and sample handling   

**Figure 7:**
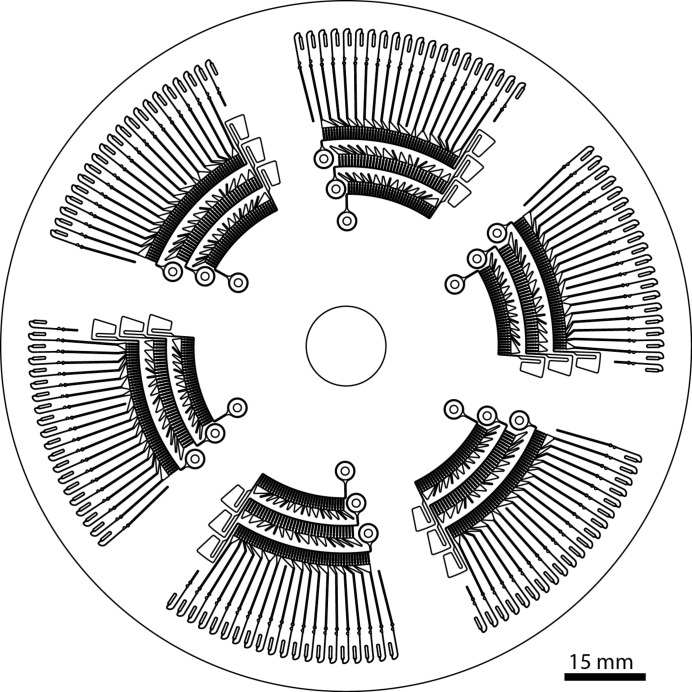
Layout of the SAXS disc microfluidic chip.

The SAXS disc is a flat compact-disc-sized sample environment used for the handling and mixing of microlitre quantities of sample. The disc is divided into identical and independent sextants that each constitute an individual sample screen (Fig. 7 ▶). Each sextant has three small reservoirs for depositing solutions: 2 µl of sample, 3 µl of mixing buffer and 3 µl of screening buffer. Once the samples and buffers have been loaded onto their respective reservoirs using standard pipetting, the disc is loaded into a centrifuge device that spins the disc with a pre-programmed rotation speed pattern in two successive phases. During the first phase, the disc is rotated at a moderate frequency (20 Hz), the centrifugal force drives the liquids from the loading reservoirs through air-tight microfluidic channels and splits the solution into 120 aliquots of 40 nl each. The solutions are held in place in the aliquoting chambers by surface tension across the narrow exit of a capillary valve (Cho *et al.*, 2007 ▶). After 90 s, all of the aliquoting chambers are filled and the rotation frequency increases (the second phase, 150 Hz for 5 min). The increased centrifugal force overcomes the capillary forces in the capillary valves and the liquid in the aliquots can further flow in one of the 20 air-tight cylindrical measurement cells (320 µm diameter, 1 mm path length). The windows through which the X-ray beam enters and exits the sample are made of polystyrene, which has a low scattering background. Each of the chambers is filled with six aliquots that are either sample, buffer or screening buffer, and the proportion of each solution in the obtained mixtures allows one to screen a broad range of conditions (Table 1 ▶). The 20 screening conditions per sextant are obtained after spinning that takes less than seven minutes, after which the disc is transferred to the beamline for the in-air SAXS measurements.

**Figure 8:**
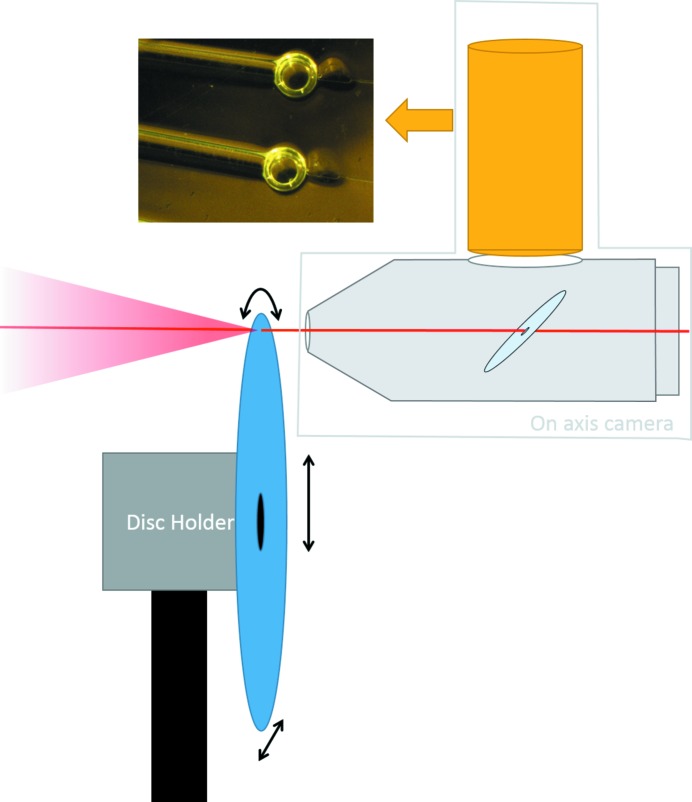
Schematic diagram of the sample environment for the microfluidic disc. The disc (blue) is mounted on a motorized disc holder that can rotate the disc and translate it in vertical and horizontal directions for fine alignment. In the on-axis microscope, the camera (in yellow) points toward a mirror (light blue) inclined at 45° and provide images of the disc in the direction of the beam, without parallax error. A hole was drilled through the mirror to let the X-ray beam (red) go through, and the mirror is installed in vacuum to reduce air absorption and scattering.

The SAXS disc is installed and aligned using a dedicated motorized sample holder and an on-axis camera equipped with a microscope for visualization of the measurement cells in the direction of the X-ray beam (Fig. 8 ▶). The sample holder has a rotation stage to bring the selected cell into the beam position and fine adjustments are performed using the *x*–*y* translation stage. In the present prototype setup, a manual alignment aided by computer-controlled motors takes approximately 30–60 s per cell, *i.e.* approximately the same time required for the batch-mode loading and washing procedures used for the standard robotic sample changer. Automatic alignment based on image processing is under development and will further reduce the time necessary to rotate and align the SAXS disc for high-throughput screening.

#### 4.2.2. SAXS disc case study: unfolding of ribonuclease A   

SAXS data were obtained from urea-induced denaturation states of ribonuclease A (RNase A) using the SAXS disc. The unfolding of RNase A has been well characterized (Pace *et al.*, 1990 ▶; Sosnick & Trewhella, 1992 ▶) and the protein is thus a suitable standard to test the efficiency of the disc, both as a tool for mixing samples and as a sample environment for SAXS data collection. The appropriate reservoirs were loaded with 2 µl of unfolded RNase A solution (25 mg ml^−1^, in 6 *M* urea, formate 30 m*M*, pH 3.5), 3 µl of 6 *M* of a matched urea buffer (formate 30 m*M*, pH 3.5) and 3 µl of a refolding buffer (no urea, formate 30 m*M*, pH 3.5). After spinning, 18 of the 20 measurement cells were filled with the sample; two chambers did not fill because of the presence of bubbles. SAXS data were collected using a 120 × 200 µm incident beam with 50 ms exposure times. The reduced and subtracted scattering profiles and the extracted *R*_g_ values were directly compared with the measurements using the sample-changer robot in batch mode (Fig. 6 ▶). Overall, the SAXS data collected from the SAXS disc and the standard SAXS setup agree well, although the data derived from the disc have lower signal-to-noise ratios. It should be noted that the sample changer measurements required 100 times more sample to complete the same experiment and more than 30 min of preparation time was spent to manually set up the urea dilution series.

**Figure 9:**
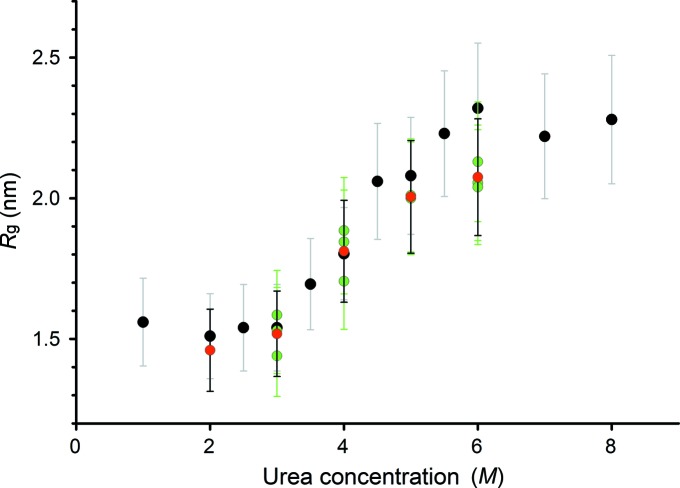
Evolution of the radius of gyration of ribonuclease A as a function of urea concentration. *R*_g_ values computed from the sample-changer curves and from the SAXS disc are shown in black and in green for different protein concentrations (red points depict the average *R*_g_ values).

The effects of urea on the structure of RNase A are reflected in the increase in *R*_g_. The *R*_g_ value could only be determined here with relatively high uncertainties because RNase A is prone to radiation damage and only the first frame of 50 ms was used for analysis. The *R*_g_ value increases from 1.5 nm to a plateau value of 2.2 nm as the concentration of urea increases (Fig. 9 ▶). This observation is consistent with *R*_g_ values reported in the literature (Sosnick & Trewhella, 1992 ▶) and indicate that the protein undergoes a transition from a compact folded state to multiple unfolded states upon urea denaturation. At low urea concentrations, the experimental intensities both from the SAXS disc measurements and from the sample changer fit the intensities calculated from the crystal structure of RNase A as shown in Fig. 8 ▶ [PDB code 1fs3 (Chatani *et al.*, 2002 ▶), χ^2^ = 0.61 and 0.88 for SAXS disc and sample changer measurements, respectively]. The consistency between the SAXS data collected from the SAXS disc *versus* those data collected from the sample changer shows that the integrated microfluidics of the disc function as an efficient mixing platform to deliver samples in the correct ratios to the measurement cells.

Although the SAXS disc data are of somewhat reduced quality compared with those collected with the sample changer, the disc data are still very much interpretable. Importantly, the SAXS disc affords an opportunity to rapidly screen multiple sample conditions using very low volumes. Consequently, for high-throughput screening of solution additives, this microfluidic device offers an attractive alternative to standard SAXS data collection strategies. The SAXS disc is currently being integrated into an automated low sample consumption system for rapid screening.

## 5. Automation of SAXS experiment and data analysis   

Efficient work with the robotic sample changer at P12 requires full automation of the SAXS experiment and its analysis. Synchronized and automated sample loading, data collection, reduction, processing, analysis and modelling protocols are needed to improve the overall quality assurance and simplify user operations. A typical SAXS experiment utilizing the in-vacuum setup and the sample-changer robot typically requires a repetition of a well defined sequence of data collection steps. Each measurement comprises the following steps: (*a*) cleaning of the capillary where the specimen is exposed to X-rays; (*b*) transport of the specimen into the capillary; (*c*) opening of the shutter; (*d*) flow of the specimen through the capillary to minimize radiation damage; (*e*) collection of the scattering pattern; (*f*) recording of monitor values, such as transmitted beam intensity, capillary temperature *etc.*; (*g*) closing of the shutter; and (*h*) removal of the exposed specimen from the capillary. After the sample loading and data collection steps have been completed, the data should be automatically processed. Automation of these procedures, especially when considering that thousands of data sets can be produced during a user shift, is critical for the efficiency, reliability and outcomes of the experiments.

### 5.1. Automation of the SAXS experiments with a sample-changer robot   

Automated collection of SAXS data requires synchronized and coordinated actions of all beamline components. Reliable communication between the devices at P12 is based on the threefold integrated networking environment (Bartkiewicz & Duval, 2007 ▶). This environment provides a programming interface to implement servers controlling the hardware devices over the network. These devices include the automatic sample changer, a beam shutter, an X-ray detector and various monitors such as those for transmitted beam intensity, storage ring current, pressure inside the fly tube and temperature of the capillary *etc.* To automate the measurement, the sequence of actions needs to run autonomously. This task is executed by the BMS, a key component of the automated SAXS experiment, first implemented at the EMBL X33 beamline (Franke *et al.*, 2012 ▶).

**Figure 10:**
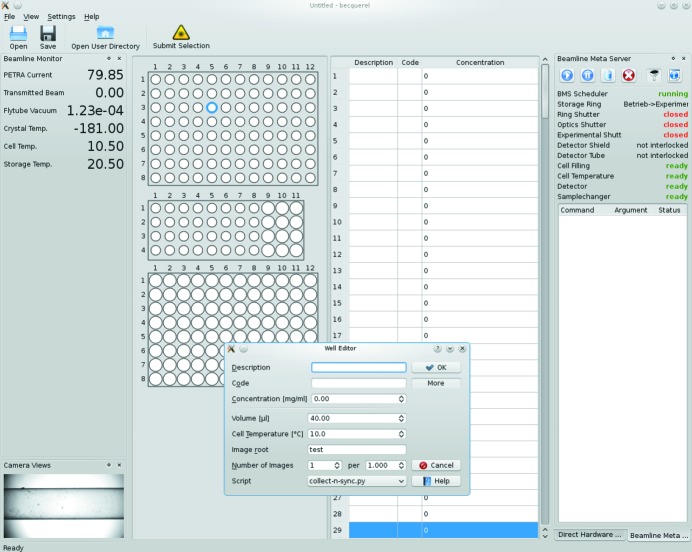
A screenshot of the graphical user interface of the P12 beamline.

The role and the importance of the BMS are illustrated by describing a typical measurement and the actions carried out by the user. The user places the macromolecular solutions and respective buffers in the sample changer tray. Using the graphical user interface *BECQUEREL* (Fig. 10 ▶), the user enters the positions of the specimens in the tray and their details, including description, code, concentration, temperature, volume, desired number of frames to be collected and exposure time per frame. This can be done either in advance or shortly before the measurement. The user determines the order in which the specimens should be measured. *BECQUEREL* allows the samples with their corresponding buffers based on the code to be associated; in this case the order of the measurements will be determined semi-automatically. From this point the BMS is programmed to perform the sequence of the hardware actions necessary for the desired outcome and no further manual intervention is needed. The BMS measuring interface allows for attended, unattended and remote operation, and the latter option, which was already in use at X33, is routinely offered to the users of P12.

### 5.2. Optimization of SAXS measurements   

The SAXS measurement is usually performed according to either of two scenarios. (1) The specimen is loaded into the capillary and does not move during the exposure. This way a volume down to only 5 µl can be measured but the static sample is subject to radiation damage. (2) The specimen is loaded and then pumped through the capillary such that the flow, the X-ray exposure and data collection are started simultaneously. Subsequently, the specimen flow stops, the beam is turned off and data collection stops at the same time. The exposure of the moving specimen reduces radiation damage but requires a higher sample volume (typically 20–40 µl). Owing to the network overhead, the synchronization between start and stop of the exposure, data collection and movement of the specimen may not be precise. In reality, up to 50% of the loaded volume might not be exposed to X-rays and, thus, is wasted from the experimenter’s point of view. Moreover, a lack of synchronization may lead to a situation where the shutter is open, data are collected but the flow of the specimen has not yet started. Alternatively, the specimen flow may stop before the exposure and data collection are finished. Both of these cases can lead to local radiation damage of the specimen.

To overcome this lack of synchronization, we introduced and tested a new setup where exposure starts with an empty capillary and the specimen flow through the capillary is initiated only once multi-frame data collection has started. The volume flowed during exposure is chosen to be larger than the sample volume such than the exposure ends again with an empty capillary. After data collection, the sample frames are distinguished from the empty capillary frame using the same algorithms as for detection of radiation damage (see below). This mode of operation ensures that 100% of the specimen volume is exposed to the X-ray beam and the flow of the specimen is guaranteed during the entire exposure (thus minimizing radiation damage). This setup does not require exact synchronization between data collection, specimen exposure and flow in the capillary and significantly improves the reproducibility of the measurements.

### 5.3. Automated data processing and analysis   

**Figure 11:**
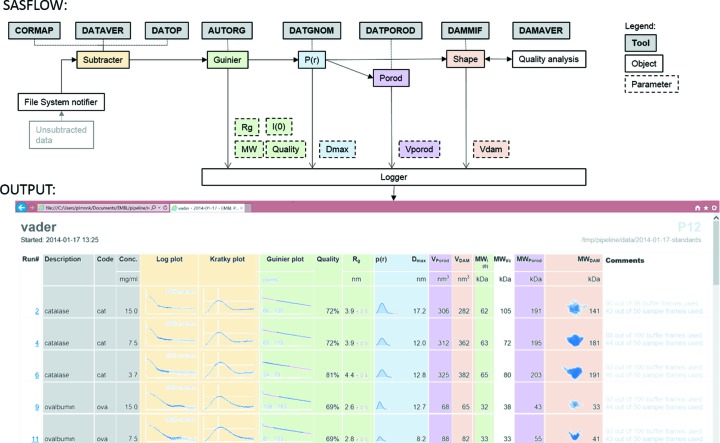
Schematic overview (top) of the integrated modules of the P12 automated data processing pipeline. See text for further details. Screenshot (bottom) of the pipeline summary table.

Modern SAXS beamlines are capable of producing large amounts of data and automated processing and analysis is nowadays a necessary prerequisite for an efficient beamline operation. The first prototype pipeline encompassing the entire data analysis process was developed for the X33 beamline (Franke *et al.*, 2012 ▶). On P12, a full data analysis pipeline (SASFLOW), starting from handling two-dimensional scattering patterns and ending with the construction of the *ab initio* low-resolution particle shape, is implemented, which is a further development of the prototype reported by Franke *et al.* (2012 ▶). Another major advantage of the modular setup of SASFLOW is the possibility to easily add additional components to the pipeline for specific experiment types. For example, a number of customized modules including buffer recognition and correlation of biophysical data with SAXS data were added for the on-the-fly data processing and analysis of the on-line chromatography SEC–SAXS (TDA) experiments. The automated analysis using the SEC–SAXS data in combination with TDA is still a work in progress. Below we shall briefly present the main features of SASFLOW, highlighting the recent developments made when implementing the pipeline to P12 (Fig. 11 ▶).

The outcome of a SAXS measurement is a two-dimensional scattering pattern, which is radially averaged and normalized against the transmitted beam intensity. The radial averaging previously was done by the BMS and tailored to the particular setup; at P12, it has been made hardware independent. This allows reproduction of the radial averaging step by the user.

Exposure of a specimen results in a number of frames (typically 20), which are compared to detect radiation damage using a novel approach called correlation map (Franke *et al.*, submitted). The frames that do not have any statistically significant differences are automatically averaged to improve the signal-to-noise ratio. Typically a buffer is measured before and after each sample. The buffer background is subtracted from the sample signal, followed by normalization by the sample concentration.

Further analysis of the subtracted data includes evaluation of the radius of gyration (*R*_g_) and the forward scattering *I*(0) from the Guinier approximation (Guinier & Fournet, 1955 ▶). The forward scattering *I*(0) as well as the Porod volume are used to estimate the molecular weight of the macromolecule. Currently, molecular weights are determined from the forward scattering value using a calibration standard (bovine serum albumin). Automatic absolute scaling, using the scattering of pure water and of the empty capillary, is in development and will be available in the near future. The pair distance distribution function *p*(*r*) is automatically computed and provides another estimate for the radius of gyration and the maximum dimension of the particle, *D*_max_. The particle shape is then automatically reconstructed *ab initio* using *DAMMIF* (Franke & Svergun, 2009 ▶).

The derived overall parameters are stored in an XML summary file, which can be visualized by a web browser (Fig. 11 ▶ *b*). To provide real-time visual feedback a number of plots have been included into the P12 summary. The experimental data are plotted as log *I*(*s*) *versus s*. The degree of compactness of the macromolecule is conveniently represented as a Kratky plot *s* ^2^ *I*(*s*) *versus s*: a bell-shaped peak indicates a globular particle, whereas a plateau is typical for extended or unfolded proteins. Prior to plotting, the intensities are divided by *I*(0) and *s* is multiplied by *R*_g_ in order to obtain the normalized (or dimensionless) Kratky plot. In the case of a globular particle the peak position would be at 3^1/2^, with a maximum around 1.104. Linearity of the Guinier plot 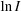
(*s*) *versus s* ^2^ is a sensitive indicator of the quality of the experimental data. Deviations from linearity point to interparticle interference effects or polydispersity of the sample. The distance distribution function *p*(*r*) is plotted to scale alongside the *ab initio* model. Presenting such a summary to the user in real time gives the possibility of optimizing the sample measurement conditions and repeating the measurement.

## 6. Conclusion   

The recent tremendous progress in biological applications of solution scattering has significantly increased the demand for high-quality biological SAXS at synchrotron facilities, and many of the new users from the biological community attracted to this technique are novices to the field. This fact imposes, in addition to the technical requirements of high beam brilliance, stability and low background, the needs for rapid and robust autonomous operation and for simplicity of use of the beamline environment. With the new influx of users, biological solution SAXS beamlines can no longer afford to operate in manual modes like they did a decade ago.

We described here a versatile P12 beamline setup optimized for solution scattering studies combining high brilliance and low background and dedicated sample environments including a solution handling robot, on-line sample purification and characterization, and extended options for time-resolved and microfluidic high-throughput studies. A robust and efficient automation of the data collection and handling allows for SAXS data collection and analysis with minimal intervention from the experimentalist with the preliminary results provided within a few minutes. With this automation not only can the experimentalists measure many more samples during a visit to the synchrotron, but the direct feedback provided by the data analysis pipeline allows for a more interactive approach, *e.g.* determining that a sample needs to be re-measured or its buffer conditions must be adjusted. Moreover, mail-in and remote operation are routinely offered to users. For mail-in experiments, the samples sent by the users are measured by the beamline staff, and the raw and processed data and preliminary *ab initio* models are sent back to the user. For remote operation, the samples sent by the user are placed in the sample changer storage tray by the beamline staff. The user takes full control of the measurements over the internet using the free NX client of NoMachine (http://www.nomachine.com), as described by Franke *et al.* (2012 ▶), and the data collection is performed from home or office (for more information see: http://www.embl-hamburg.de/biosaxs/user_info.html).

The instrumental setup presented here fulfils the expectations of most biological SAXS users but the instrument is of course being further developed. Thus, an environment for SAXS experiments in extreme conditions (chemical, temperature, pressure) is being prepared in collaboration with HZG. Among the ongoing developments at P12 are the extended possibilities for rapid kinetic experiments, which will be accessible as of spring 2015 thanks to the multilayer monochromator (presently being installed) that will boost the flux by a factor of 50. To allow for a sub-microsecond range time resolution, appropriate devices are being developed to produce and control precise X-ray pulses, to synchronize the measurements, and to achieve a rapid triggering of the biological reactions. The use of a microfluidics chip on the SAXS beamline is also being further developed. For the screening chip, automatic alignment of the well with the beam is being implemented to allow full automation of the data collection.

## Figures and Tables

**Table 1:** Proportion of screening buffer and protein concentration in the measurement chamber of the SAXS disc

	0%	16.6%	33.3%	50%	66.6%
*C*_0_ †	#1				
*C*_0_/2	#13	#5	#17	#9	
*C*_0_/3	#11	#16	#8	#4	#19
*C*_0_/6	#7	#10	#18	#15	#3
0	#20	#6	#14	#2	#12

† *C*_0_ is the concentration of the initial solution pipetted on to the disc.

## References

[bb1] Bartkiewicz, P. & Duval, P. (2007). *Meas. Sci. Technol.* **18**, 2379–2386.

[bb2] Blanchet, C. E., Hermes, C., Svergun, D. I. & Fiedler, S. (2015). *J. Synchrotron Rad.* **22**, 461–464. 10.1107/S160057751402829X PMC4344362 25723949

[bb3] Blanchet, C. E. & Svergun, D. I. (2013). *Annu. Rev. Phys. Chem.* **64**, 37–54. 10.1146/annurev-physchem-040412-110132 23216378

[bb4] Blanchet, C. E., Zozulya, A. V., Kikhney, A. G., Franke, D., Konarev, P. V., Shang, W., Klaering, R., Robrahn, B., Hermes, C., Cipriani, F., Svergun, D. I. & Roessle, M. (2012). *J. Appl. Cryst.* **45**, 489–495.

[bb5] Callender, R. & Dyer, R. B. (2006). *Chem. Rev.* **106**, 3031–3042. 10.1021/cr050284b 16895316

[bb101] Chatani, E., Hayashi, R., Moriyama, H. & Ueki, T. (2002). *Protein Sci.* **11**, 72–81. 10.1110/ps.31102 PMC2368775 11742124

[bb6] Cho, H., Kim, H. Y., Kang, J. Y. & Kim, T. S. (2007). *J. Colloid Interface Sci.* **306**, 379–385. 10.1016/j.jcis.2006.10.077 17141795

[bb7] Chu, B. & Hsiao, B. S. (2001). *Chem. Rev.* **101**, 1727–1762. 10.1021/cr9900376 11709997

[bb8] Classen, S., Rodic, I., Holton, J., Hura, G. L., Hammel, M. & Tainer, J. A. (2010). *J. Synchrotron Rad.* **17**, 774–781. 10.1107/S0909049510028566 PMC2964114 20975223

[bb9] David, G. & Pérez, J. (2009). *J. Appl. Cryst.* **42**, 892–900.

[bb10] Dubuisson, J.-M., Decamps, T. & Vachette, P. (1997). *J. Appl. Cryst.* **30**, 49–54.

[bb11] Ducrée, J., Haeberle, S., Lutz, S., Pausch, S., von Stetten, F. & Zengerle, R. (2007). *J. Micromech. Microeng.* **17**, S103–S115.

[bb12] Ellis, P. J., Cohen, A. E. & Soltis, S. M. (2003). *J. Synchrotron Rad.* **10**, 287–288. 10.1107/s0909049503003285 12714764

[bb13] Fernández, M., Keyriläinen, J., Serimaa, R., Torkkeli, M., Karjalainen-Lindsberg, M. L., Tenhunen, M., Thomlinson, W., Urban, V. & Suortti, P. (2002). *Phys. Med. Biol.* **47**, 577–592. 10.1088/0031-9155/47/4/303 11900192

[bb14] Fislage, M., Brosens, E., Deyaert, E., Spilotros, A., Pardon, E., Loris, R., Steyaert, J., Garcia-Pino, A. & Versionées, W. (2014). *Nucleic Acids Res.* pp. 1–15. 10.1093/nar/gku213 PMC4027165 24634441

[bb15] Franke, D., Kikhney, A. G. & Svergun, D. I. (2012). *Nucl. Instrum. Methods Phys. Res. Sect. A*, **689**, 52–59.

[bb16] Franke, D. & Svergun, D. I. (2009). *J. Appl. Cryst.* **42**, 342–346. 10.1107/S0021889809000338 PMC5023043 27630371

[bb17] Fratzl, P. (2003). *J. Appl. Cryst.* **36**, 397–404.

[bb18] Glatter, O. & Kratky, O. (1982). *Small-Angle X-ray Scattering.* London: Academic Press.

[bb19] Graceffa, R., Nobrega, R. P., Barrea, R. A., Kathuria, S. V., Chakravarthy, S., Bilsel, O. & Irving, T. C. (2013). *J. Synchrotron Rad.* **20**, 820–825. 10.1107/S0909049513021833 PMC3795536 24121320

[bb20] Graewert, M. A. & Svergun, D. I. (2013). *Curr. Opin. Struct. Biol.* **23**, 748–754. 10.1016/j.sbi.2013.06.007 23835228

[bb21] Guinier, A. & Fournet, G. (1955). *J. Polym. Sci.* **1**, 268.

[bb22] Henrich, B., Bergamaschi, A., Broennimann, C., Dinapoli, R., Eikenberry, E. F., Johnson, I., Kobas, M., Kraft, P., Mozzanica, A. & Schmitt, B. (2009). *Nucl. Instrum. Methods Phys. Res. Sect. A*, **607**, 247–249.

[bb23] Jeffries, C. M., Graewert, M. A., Svergun, D. I. & Blanchet, C. E. (2015). *J. Synchrotron Rad.* **22**, 273-279. 10.1107/S1600577515000375 25723929

[bb24] Kathuria, S. V., Guo, L., Graceffa, R., Barrea, R., Nobrega, R. P., Matthews, C. R., Irving, T. C. & Bilsel, O. (2011). *Biopolymers*, **95**, 550–558. 10.1002/bip.21628 PMC3278217 21442608

[bb25] Kirby, N. M., Mudie, S. T., Hawley, A. M., Cookson, D. J., Mertens, H. D. T., Cowieson, N. & Samardzic-Boban, V. (2013). *J. Appl. Cryst.* **46**, 1670–1680.

[bb26] Kozin, M. B. & Svergun, D. I. (2001). *J. Appl. Cryst.* **34**, 33–41.

[bb27] Labrador, A., Cerenius, Y., Svensson, C., Theodor, K. & Plivelic, T. (2013). *J. Phys. Conf. Ser.* **425**, 72019.

[bb28] Lafleur, J. P., Snakenborg, D., Nielsen, S. S., Møller, M., Toft, K. N., Menzel, A., Jacobsen, J. K., Vestergaard, B., Arleth, L. & Kutter, J. P. (2011). *J. Appl. Cryst.* **44**, 1090–1099.

[bb29] Li, Y., Beck, R., Huang, T., Choi, M. C. & Divinagracia, M. (2008). *J. Appl. Cryst.* **41**, 1134–1139.

[bb30] Lindorff-Larsen, K., Piana, S., Dror, R. O. & Shaw, D. E. (2011). *Science*, **334**, 517–520. 10.1126/science.1208351 22034434

[bb31] Lipfert, J. & Doniach, S. (2007). *Annu. Rev. Biophys. Biomol. Struct.* **36**, 307–327. 10.1146/annurev.biophys.36.040306.132655 17284163

[bb32] Lipfert, J., Millett, I. S., Seifert, S. & Doniach, S. (2006). *Rev. Sci. Instrum.* **77**, 046108.

[bb33] Martel, A., Burghammer, M., Davies, R., DiCola, E., Panine, P., Salmon, J. B. & Riekel, C. (2008). *Biomicrofluidics*, **2**, 024104. 10.1063/1.2943732 PMC2719262 19693407

[bb34] Martin, H. P., Brooks, N. J., Seddon, J. M., Terrill, N. J., Luckham, P. F., Kowalski, A. J. & Cabral, J. T. (2010). *J. Phys. Conf. Ser.* **247**, 012050.

[bb35] Mathew, E., Mirza, A. & Menhart, N. (2004). *J. Synchrotron Rad.* **11**, 314–318. 10.1107/S0909049504014086 15211037

[bb36] Nielsen, S. S., Møller, M. & Gillilan, R. E. (2012). *J. Appl. Cryst.* **45**, 213–223. 10.1107/S0021889812000957 PMC3325496 22509071

[bb37] Nölting, B. (2005). *Protein Folding Kinetics: Biophysical Methods.* Berlin, Heidelberg: Springer.

[bb38] Pace, C. N., Laurents, D. V. & Thomson, J. A. (1990). *Biochemistry*, **29**, 2564–2572. 10.1021/bi00462a019 2110472

[bb39] Paris, O., Zizak, I., Lichtenegger, H., Roschger, P., Klaushofer, K. & Fratzl, P. (2000). *Cell. Mol. Biol.* **46**, 993–1004. 10976879

[bb40] Pernot, P. *et al.* (2013). *J. Synchrotron Rad.* **20**, 660–664. 10.1107/S0909049513010431 PMC3943554 23765312

[bb41] Roessle, M. W., Klaering, R., Ristau, U., Robrahn, B., Jahn, D., Gehrmann, T., Konarev, P., Round, A., Fiedler, S., Hermes, C. & Svergun, D. (2007). *J. Appl. Cryst.* **40**, s190–s194.

[bb42] Round, A., Brown, E., Marcellin, R., Kapp, U., Westfall, C. S., Jez, J. M. & Zubieta, C. (2013). *Acta Cryst.* D**69**, 2072–2080. 10.1107/S0907444913019276 24100325

[bb43] Round, A., Felisaz, F., Fodinger, L., Gobbo, A., Huet, J., Villard, C., Blanchet, C. E., Pernot, P., McSweeney, S., Roessle, M., Svergun, D. I. & Cipriani, F. (2015). *Acta Cryst.* D**71**, 67–75. 10.1107/S1399004714026959 PMC4304687 25615861

[bb44] Round, A. R., Franke, D., Moritz, S., Huchler, R., Fritsche, M., Malthan, D., Klaering, R., Svergun, D. I. & Roessle, M. (2008). *J. Appl. Cryst.* **41**, 913–917. 10.1107/S0021889808021018 PMC4233401 25484841

[bb45] Sosnick, T. R. & Trewhella, J. (1992). *Biochemistry*, **31**, 8329–8335. 10.1021/bi00150a029 1525171

[bb46] Spilotros, A. & Svergun, D. I. (2014). *Encyclopedia of Analytical Chemistry*, edited by R. A. Meyers. Chichester: John Wiley and Sons.

[bb100] Stein, P. E., Leslie, A. G., Finch, J. T. & Carrell, R. W. (1991). *J. Mol. Biol.* **221**, 941–959. 10.1016/0022-2836(91)80185-w 1942038

[bb47] Stuhrmann, H. (1980). *Synchrotron Radiation Research*, edited by H. Winick & S. Doniach, pp. 513–531. New York: Plenum Press.

[bb48] Svergun, D. I. (1999). *Biophys. J.* **76**, 2879–2886. 10.1016/S0006-3495(99)77443-6 PMC1300260 10354416

[bb49] Svergun, D., Barberato, C. & Koch, M. H. J. (1995). *J. Appl. Cryst.* **28**, 768–773.

[bb50] Trebbin, M., Steinhauser, D., Perlich, J., Buffet, A., Roth, S. V., Zimmermann, W., Thiele, J. & Förster, S. (2013). *Proc. Natl Acad. Sci. USA*, **110**, 6706–6711. 10.1073/pnas.1219340110 PMC3637725 23569240

[bb51] Vestergaard, B., Groenning, M., Roessle, M., Kastrup, J. S., Van De Weert, M., Flink, J. M., Frokjaer, S., Gajhede, M. & Svergun, D. I. (2007). *PLoS Biol.* **5**, 1089–1097. 10.1371/journal.pbio.0050134 PMC1858711 17472440

[bb52] Volkov, V. V. & Svergun, D. I. (2003). *J. Appl. Cryst.* **36**, 860–864.

